# Absence of progesterone receptor membrane component 1 reduces migration and metastasis of breast cancer

**DOI:** 10.1186/s12964-021-00719-w

**Published:** 2021-04-08

**Authors:** Sang R. Lee, Young Ho Lee, Seong Lae Jo, Jun H. Heo, Globinna Kim, Geun-Shik Lee, Beum-Soo An, In-Jeoung Baek, Eui-Ju Hong

**Affiliations:** 1 College of Veterinary Medicine, Chungnam National University, Suite 401, Veterinary Medicine Bldg., 99, Daehak-ro, Yuseong-gu, Daejeon, 34134 Republic of Korea; 2 Department of Convergence Medicine, Asan Medical Center, University of Ulsan College of Medicine, 88, Olympic-ro 43-gil, Songpa-gu, Seoul, 05505 Republic of Korea; 3 College of Veterinary Medicine, Kangwon National University, Chuncheon, 24341 Republic of Korea; 4 Department of Biomaterials Science, Pusan National University, Miryang, 50463 Republic of Korea

**Keywords:** Pgrmc1, Breast cancer, Lung, Metastasis, Migration

## Abstract

**Background:**

Progesterone receptor membrane component 1 (Pgrmc1) is a non-classical progesterone receptor associated with the development of the mammary gland and xenograft-induced breast cancer. Importantly, Pgrmc1 is associated with the expression of estrogen receptor alpha and can be used for predicting the prognosis of breast cancer. Whether the genetic deletion of Pgrmc1 affects the progression of breast cancer is still unclear.

**Methods:**

We used MMTV-PyMT transgenic mice that spontaneously develop breast tumors. In backcrossed FVB *Pgrmc1* knockout (KO) mice, we monitored the development of the primary tumor and lung metastasis. In MCF-7 and MDA-MB-231 tumor cell lines, the migratory activity was evaluated after *Pgrmc1* knockdown.

**Results:**

There was no significant difference in the development of breast cancer in terms of tumor size at 13 weeks of age between WT and *Pgrmc1* KO mice. However, *Pgrmc1* KO mice had a significantly longer survival duration compared with WT mice. Furthermore, *Pgrmc1* KO mice exhibited a significantly lower degree of lung metastasis. Compared with those of WT mice, the tumors of *Pgrmc1* KO mice had a low expression of focal adhesion kinase and epithelial–mesenchymal transition markers. *PGRMC1* knockdown resulted in a significantly reduced migration rate in breast cancer cell lines.

**Conclusions:**

*Pgrmc1* KO mice with breast cancer had a prolonged survival, which was accompanied by a low degree of lung metastasis. PGRMC1 showed a significant role in the migration of breast cancer cells, and may serve as a potential therapeutic target in breast cancer.

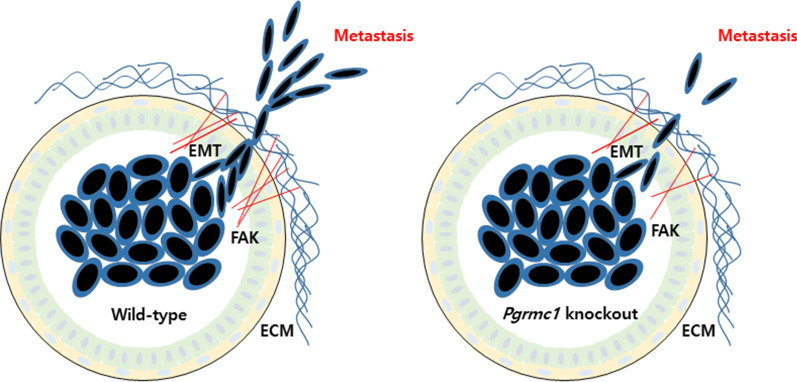

**Video Abstract**

**Supplementary Information:**

The online version contains supplementary material available at 10.1186/s12964-021-00719-w.

## Background

Breast cancer is the most prevalent malignancy in women, has a high mortality rate, and has a diverse range of epidemiological factors including genetic and environmental causes [1]. While the rate of early diagnosis of breast cancer has improved, the recurrence of breast cancer is still difficult to control and results in a poor survival rate [2]. During the metastatic process, epithelial tumor cells leave the breast tumor nodule, migrate to a new tissue site, and reform as a secondary tumor nodule. The typical phenotype of metastasis is the induction of epithelial–mesenchymal transition (EMT), including cadherin switches, and a positive correlation between Vimentin and Slug expression [3]. Although metastasis occurs in various organs such as the liver and the brain, more than half of the mortality in breast cancer occurs in those with lung metastasis [4, 5].

In breast cancer, conventional markers including estrogen receptor alpha (ERα) and progesterone receptor (PR), are indicators for hormonal treatment, but do not act as prognostic markers [6]. Furthermore, the PR status was not significantly associated with the survival expectancy in breast cancer patients [7]. Accordingly, further study for breast cancer metastasis should be focused on factors other than hormone receptors, which are the conventional targets for breast cancer treatment.

Progesterone receptor membrane component 1 (Pgrmc1) is a non-classical progesterone receptor with diverse roles in metabolism and steroidogenesis [8, 9]. In addition, Pgrmc1 was recently identified as a regulator of mammary gland development [10]. Furthermore, considering that Pgrmc1 expression is associated with ERa expression, suppression of Pgrmc1 has been considered as a potential therapeutic approach against the progression of breast cancer [11]. As *Pgrmc1* is involved in estrogen synthesis, a recent study showed that a high level of Pgrmc1 promotes the development of breast cancer in a xenograft model [12]. Moreover, another study also reported that *Pgrmc1* is involved in mTOR activation and EGFR signaling [13]. These findings suggest that *Pgrmc1* is closely involved in the development of breast cancer; however, an in vivo study using genetic deletion of *Pgrmc1* has yet to be conducted. Furthermore, it is still unclear whether metastasis of breast cancers can be influenced by the levels of PGRMC1 protein. Particularly, the progression and metastasis of breast cancer should be evaluated in genetically engineered mice such as MMTV-PyMT [14], which were useful in investigating the role of adhesion proteins in breast cancer metastasis [15].

In the present study, we used transgenic MMTV-PyMT mice that spontaneously develop breast cancer and deleted the *Pgrmc1* gene. At 13 weeks of age, there was no significant difference in the development of breast cancer between wild-type (WT) and *Pgrmc1* knockout (KO) mice; however, *Pgrmc1* KO mice had a significantly longer survival period and a significantly lower degree of lung metastasis compared with WT mice, thus suggesting the possible role of Pgrmc1 in the metastasis of breast cancer and its potential value as a therapeutic target.

## Methods

### Animals

We used *Pgrmc1* KO mice that had been backcrossed for more than 10 generations from the C57Bl/6J background into the FVB background [16]. PyMT (FVB/N-Tg(MMTV-PyVT)634Mul/J) transgenic mice were purchased from the Jackson Laboratory (022974). The backcrossed FVB *Pgrmc1* KO mice were crossed with the PyMT FVB mice. We introduced PyMT-*Pgrmc1* male and *Pgrmc1* female for WT group or PyMT *Pgrmc1* hemizygous (KO) male and *Pgrmc1* homozygous (KO) female for KO group. In this study, we used female PyMT transgenic mice with or without Pgrmc1 that were housed in a pathogen-free facility at Chungnam National University (Daejeon, Korea) with a standard 12 light/12 dark cycle and ad libitum supply of standard chow and water. All animal experiments were approved and performed under the Chungnam Facility Animal Care Committee (202006A-CNU-105). The mice were euthanized using CO_2_ overdose.

### RNA isolation, reverse transcription, and qRT-PCR

RNA was extracted from breast cancer tissues, MCF-7 cells, and MDA-MB-231 cells by using the TRIzol Reagent, chloroform, isopropanol, and DEPC. cDNA was synthesized with 1 µg of total RNA using a reverse transcriptase kit (SG-cDNAS100, Smartgene, the United Kingdom) according to the manufacturer's protocol. Quantitative PCR (real-time PCR) was carried out using the primers listed in Table 1, Excel Taq Q-PCR Master Mix (SG-SYBR-500, Smartgene), and Stratagene Mx3000P (Agilent Technologies) equipped with a 96-well optical reaction plate. All experiments were repeated in triplicates, and mRNA values were calculated based on the cycle threshold and monitored for a melting curve.

**Table 1:** Primers used for real-time PCR

Gene name	Upper primer (5′–3′)	Lower primer (5′–3′)	Species
*E-Cadherin*	CAG GTC TCC TCA TGG CTT TGC	CTT CCG AAA AGA AGG CTG TCC	Mouse
*N-Cadherin*	AAA GCC TGG GAC GTA TGT GA	TTC TCT CGA TCC AGA CCA GC	Mouse
*Slug*	GCT CCA CTC CAC TCT CCT TT	CCA GCC CAG AGA ACG TAG AA	Mouse
*Vim*	ATG CTT CTC TGG CAC GTC TT	AGC CAC GCT TTC ATA CTG CT	Mouse
*E-Cadherin*	CGG ACG ATG ATG TGA ACA CC	TTG CTG TTG TGC TTA ACC CC	Human
*N-Cadherin*	CGG TTT CAT TTG AGG GCA CA	TTG GAG CCT GAG ACA CGA TT	Human
*SLUG*	CCT GGT TGC TTC AAG GAC AC	AGC AGC CAG ATT CCT CAT GT	Human
*VIM*	GAG TCC ACT GAG TAC CGG AG	ACG AGC CAT TTC CTC CTT CA	Human

### Western blot

Protein was extracted from breast cancer tissues, MCF7 cells, and MDA-MB-231 cells by homogenization with T-PER buffer. Protein samples were loaded in equal amounts to SDS-PAGE gels and were proceeded to electrophoresis. Gels were blotted to PVDF membranes, which were blocked and incubated with primary antibodies. After overnight incubation, the membranes were washed and incubated with secondary antibodies (211-032-171 anti-rabbit, Jackson laboratory; bs-0296G-HRP anti-mouse, Bioss). Bands were observed with ECL solution (XLS025-0000, Cyanagen) after washing three times.

The following primary polyclonal antibodies were used: rabbit anti-β-actin (sc-130656, Santa Cruz Biotechnology), PR (sc-7208, Santa Cruz), HER2 (4290, Cell Signaling Technology, CST), PCNA (13110, CST), phosphor-AKT (4060, CST), phosphor-ERK (9789, CST), PARP (9532, CST), FAK (13430, CST), rabbit monoclonal antibody to PGRMC1 (13856, CST), and mouse monoclonal antibody to ERα (sc-71064, Santa Cruz Biotechnology).

### Cell culture

All cell culture reagents were purchased from Welgene (Gyungsan, Korea). MCF-7 and MDA-MB-231 human breast cancer cells were maintained at 37 °C in a 5% CO_2_ atmosphere in DMEM (Welgene, LM001-05) supplemented with 5% (vol/vol) fetal bovine serum, penicillin (100 U/mol), and streptomycin (100 μg/ml). For *PGRMC1* knockdown, siRNA transfection was performed using the Lipofectamine 2000 reagent (11668-027, Thermo Fisher) according to the manufacturer's protocol. Negative control siRNA and *PGRMC1* siRNA #1 and #2 were purchased from Bioneer (Daejeon, Korea). The sense sequences of *PGRMC1* siRNA #1 and #2 were 5′-CAGUACAGUCGCUAGUCAA-3′ and 5′-CAGUUCACUUUCAAGUAUCA-U-3′, respectively.

### Cell migration assay

Cell migration was assessed by cell scratch assay in which the cells were scraped with micropipette tips. Measurements were performed in the same areas by drawing circles on the base of the cell culture plate. Image J (NIH, Bethesda, MD, USA) was used for analysis. Transwell plates were also used for measurements of cell migration, in which cells that migrated through the hanging insert were stained by crystal violet at the indicated times and quantified by Image J.

### Matrix metallopeptidase (MMP) measurement

MMP2/MMP9 Gel Assay Kit (Cat#: E-118GA) was purchased from Biomedical Research Service, and the measurements were carried out according to the manufacturer’s protocol.

### H&E staining

For H&E staining, slides were obtained by using 4–5 µm sections of paraffin blocks and incubated in xylene. The slides were processed for serial hydration using ethanol (100% to 70%) and tap water, and incubated with hematoxylin for 5 min. After washing with tap water for 3 min, the slides were incubated with eosin for 1 min and 20 s. The slides were then processed for serial dehydration using ethanol (70% to 100%) ethanol and xylene, and mounted with a coverglass.

### Immunofluorescence

For immunofluorescence, slides were obtained by using 4–5 µm sections of paraffin blocks and incubated overnight in xylene. The slides were then processed for serial hydration using ethanol (100% to 70%) and distilled water. Antigen retrieval was performed with 0.1% sodium citrate buffer (CA2081, Georgiachem) at 95 °C for 60 min. After cooling down, the slides were washed once with TBS-T and blocked with 3% BSA (bovine serum albumin). The slides were then incubated with the FAK and Ki67 (GeneTex, GTX16667) primary antibodies overnight at 4 °C, washed three times with TBS-T, and incubated with an anti-rabbit secondary antibody (A21207, Life Technologies) for 4 h at room temperature.

### Statistical analysis

Data are reported as mean ± standard deviation. Student's *t*-test was used to analyze the differences between the mean values of each experimental group. All statistical analyses were performed using the GraphPad Software (GraphPad Inc., San Diego, CA, USA).

## Results

### Loss of the Pgrmc1 gene extends the survival duration of breast cancer-bearing mice

**Fig. 1:**
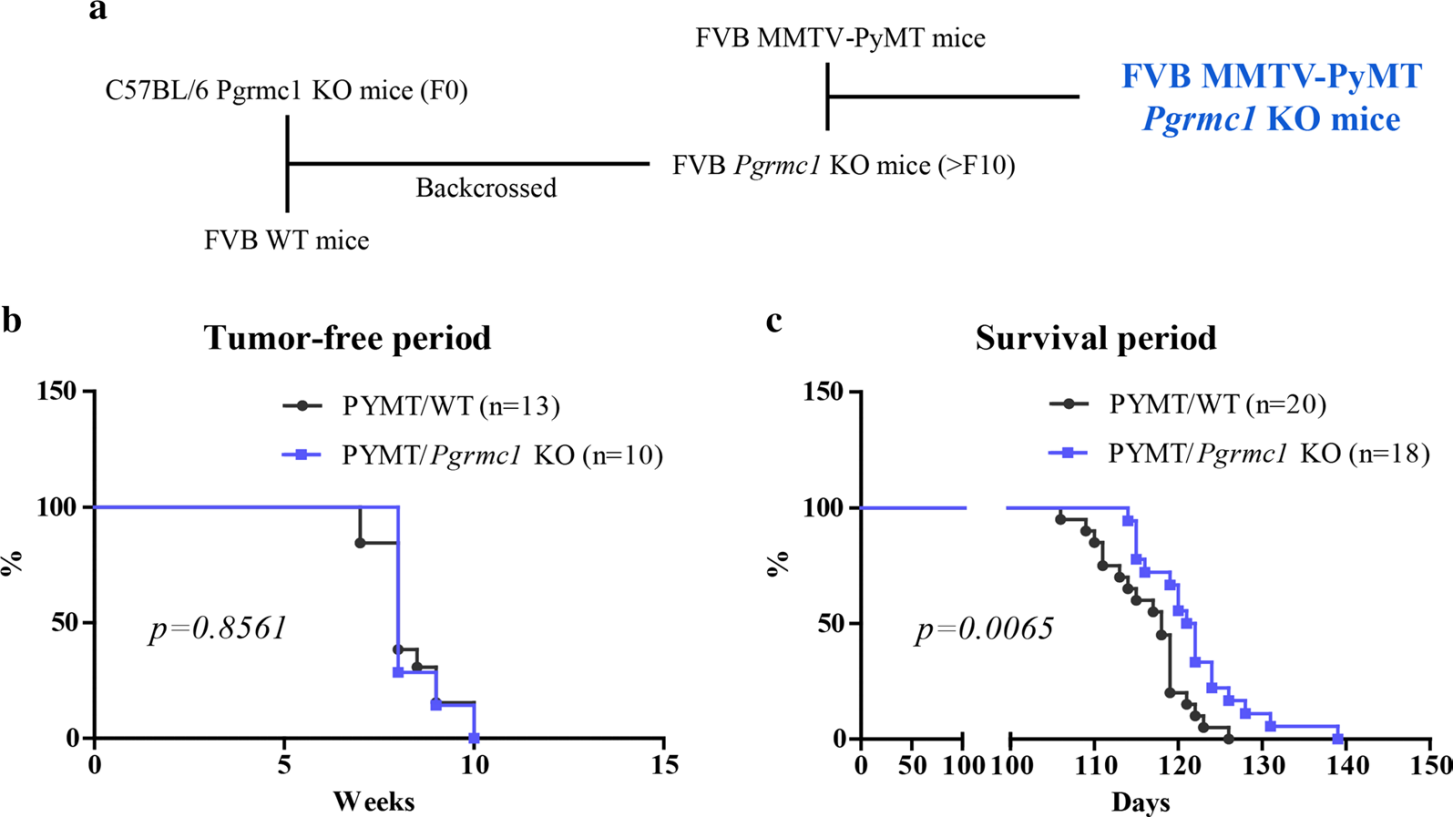
MMTV-PyMT *Pgrmc1* knockout (KO) mice with breast cancer have a longer survival period than WT mice. **a** Establishment of the FVB MMTV-PyMT *Pgrmc1* KO mice. **b** Kaplan–Meier curve of the tumor-free period according to age in weeks (WT, n = 13; KO, n = 10). **c** Kaplan–Meier curve of the total survival period according to the time of death in days (WT, n = 20; KO, n = 18)

To determine whether Pgrmc1 is involved in the induction of breast cancer, we first investigated the incidence of breast cancer in PyMT wild-type (WT) and PyMT *Pgrmc1* KO mice and their survival duration. The breeding plan for the establishment of breast cancer-bearing mice is shown in Fig. 1a. The incidence of breast cancer was determined by palpation of nodules, and there was no significant difference in the tumor-free duration between WT and *Pgrmc1* KO mice (Fig. 1b). However, tumor-bearing Pgrmc1 KO mice showed a significantly longer survival duration (*p* = 0.0065) compared with tumor-bearing WT mice, suggesting that *Pgrmc1* is related to the aggravation of the disease course of breast cancer (Fig. 1c).

### Loss of the Pgrmc1 protein does not decrease the development and proliferation of breast cancer

**Fig. 2:**
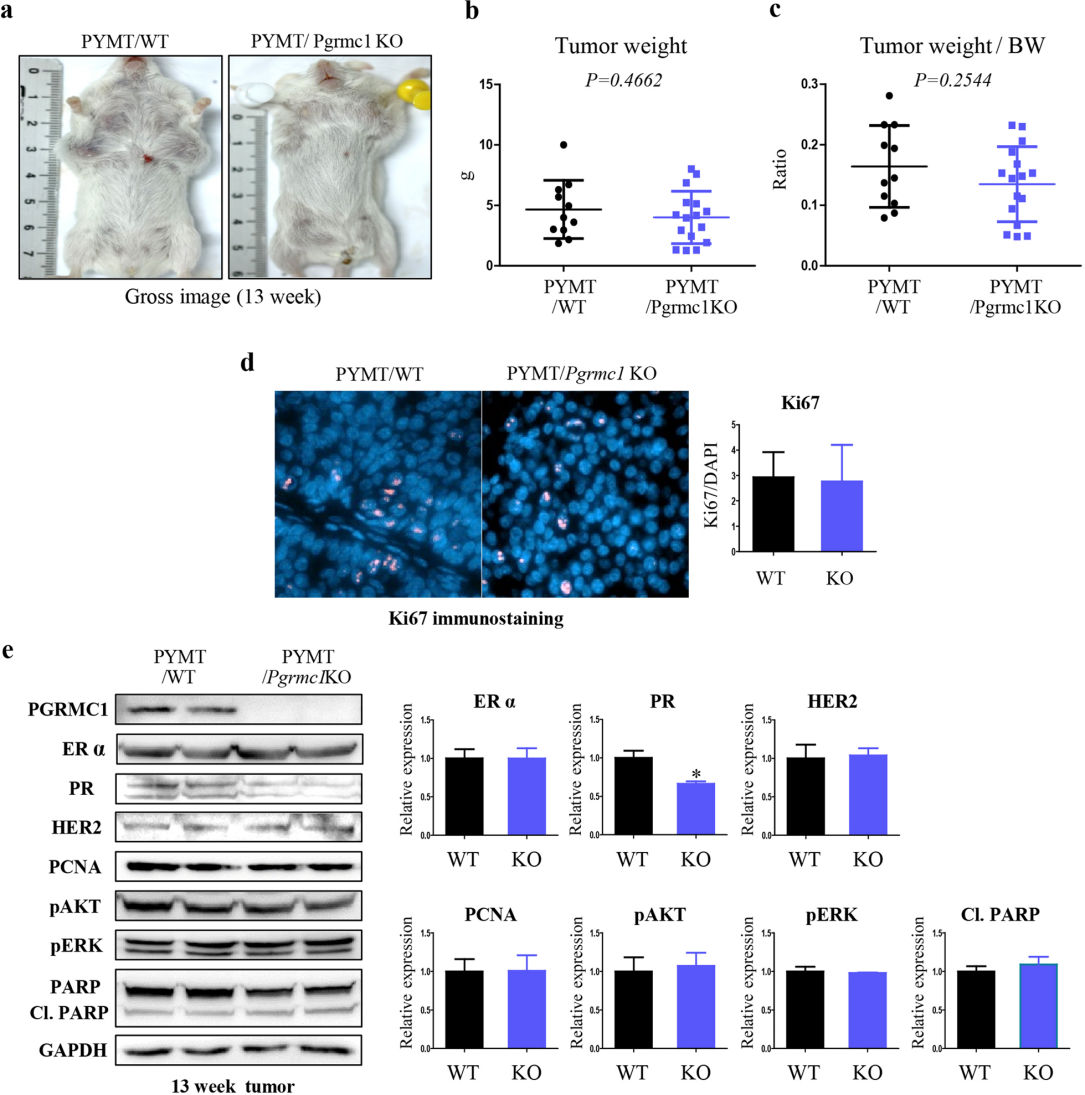
Genetic deletion of *Pgrmc1* does not suppress the growth and proliferation of breast cancer in MMTV-PyMT mice. **a** Gross images of tumor-bearing WT and *Pgrmc1* KO mice at the age of 13 weeks. **b** Tumor weight (g) of WT and *Pgrmc1* KO mice at the age of 13 weeks. **c** Tumor weight per body weight (BW) of WT and *Pgrmc1* KO mice at the age of 13 weeks. **d** Immunostaining of Ki67 in the tumors of WT and *Pgrmc1* KO mice. The number of Ki67-positive cells (pink) were counted. DAPI (blue) was used as an internal control. **e** Western blot analysis and quantification of PGRMC1, ERα, PR, HER2, PCNA, pAKT, pERK, PARP, and Cl. PARP. GAPDH was used as an internal control. Bar graphs in the right panel show the mean ± standard deviation values (WT, n = 10; KO, n = 16). *, *p* < 0.05 versus WT mice

Most mice in this study developed breast cancer at approximately 10 weeks of age; as such, we sacrificed tumor-bearing mice at 13 weeks of age to analyze the tumor development. The gross images of tumor-bearing WT and *Pgrmc1* KO mice are shown in Fig. 2a. The average weights of the tumors were not significantly different between the two groups (*p* = 0.4662; Fig. 2b). Tumor weight per body weight was lower in *Pgrmc1* KO mice, albeit the difference was not statistically significant (*p* = 0.2544; Fig. 2c). Tumor cell proliferation was assessed by using Ki67 immunostaining, but there was no significant difference between the two groups either (Fig. 2d).

The expression levels of the triple markers for breast cancer—ERα, PR, and human epidermal growth factor receptor-2 (HER2)—were analyzed in gene expression analysis. As a result, we observed that the mean expression level of PR in the tumors of Pgrmc1 mice was only 66.4% of that of WT mice (*p* < 0.05; Fig. 2e). The expression levels of genes related to proliferation (i.e., PCNA, phospho-AKT, and phospho-ERK) were not significantly different between the two groups (Fig. 2e). The expression level of cleaved PARP, a marker of apoptotic cell death, also did not show a significant difference (Fig. 2e). These results showed that despite its significant effect on survival, *Pgrmc1* does not play a strong role in tumor development.

### Migration ability and lung metastasis of breast cancer cells are reduced in Pgrmc1 KO mice

**Fig. 3:**
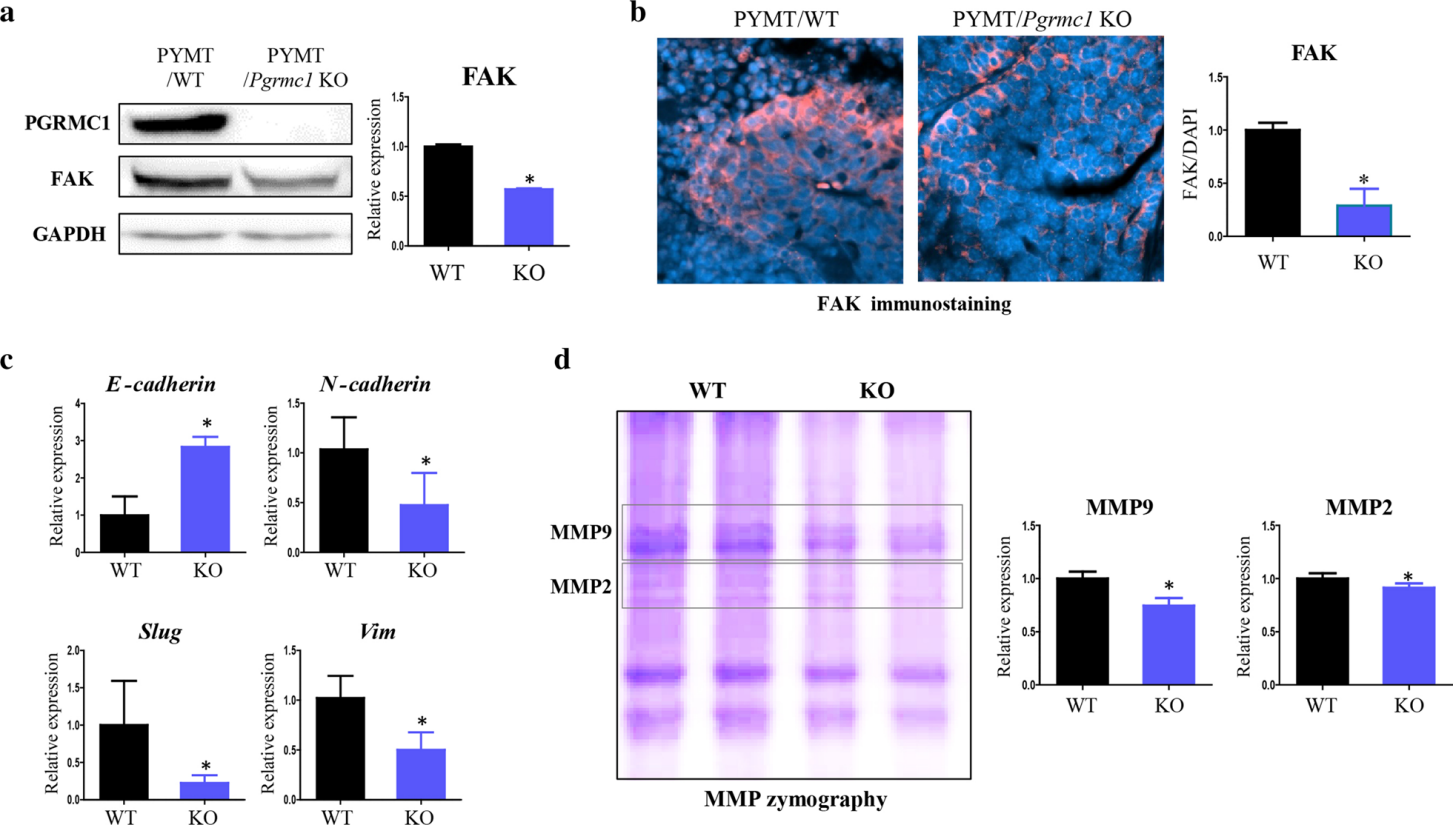
Genetic deletion of *Pgrmc1* decreases the metastatic markers in the tumors of MMTV-PyMT mice. **a** Western blot analysis and quantification of PGRMC1 and FAK in the tumors of WT and *Pgrmc1* KO mice. GAPDH was used as an internal control. **b** Immunostaining of FAK in the tumors of WT and *Pgrmc1* KO mice. The FAK-positive areas (pink) were quantified. DAPI (blue) was used as an internal control. **c** mRNA expression of an epithelial marker (*E-cadherin*) and mesenchymal markers (*N-cadherin, Slug, Vim*) in the tumors of WT and *Pgrmc1* KO mice. *Rplp0* was used as an internal control. **d** Zymographic analysis and quantification of MMP9 and MMP2 in the tumors of WT and *Pgrmc1* KO mice. Bars in the right panel show the mean ± standard deviation values (WT, n = 10; KO, n = 16). *, *p* < 0.05 versus WT mice

Considering that PGRMC1 is related to focal adhesion [17], we measured the expression of focal adhesion kinase (FAK) in our models. In Western blot, the expression of FAK was decreased by 42.9% (*p* < 0.05) in the tumors of *Pgrmc1* KO mice compared with those of WT mice (Fig. 3a). In immunostaining, the FAK expression was also decreased by 71% (*p* < 0.05) in the tumors of *Pgrmc1* KO mice (Fig. 3b).

We also measured the markers of EMT, which determines the migratory phenotype of cancer cells. The mRNA expression of the epithelial marker *E-cadherin* was increased by 2.84-fold (*p* < 0.05) in the tumors of *Pgrmc1* KO mice (Fig. 3c). Conversely, *Pgrmc1* KO mice showed lower levels of mRNA expression of the mesenchymal markers, *N-cadherin*, *Slug*, and *Vim* (*p* < 0.05, 45.9%, 22.4%, and 49.1% compared with WT mice, respectively) (Fig. 3c). These results suggest that the tumor cells of mice without Pgrmc1 would lack the mesenchymal phenotype and that their migration to other organs would be hindered.

Then, considering that tumor cells can pass through the extracellular matrix (ECM) for migration, we measured the zymographic expression of MMP9 and MMP2 that belong to the metalloproteinases family involved in the degradation of ECM and found that the expression levels of MMP9 and MMP2 in the tumors of *Pgrmc1* KO mice were decreased *(p* < 0.05, 74.6% and 91.4% compared with WT mice, respectively) (Fig. 3d). Although the reduction of MMP2 was marginal, the reductions in FAK, mesenchymal markers, and MMP9 in *Pgrmc1* KO mice may be considered as comprising a challenging environment for the migration of tumor cells.

**Fig. 4:**
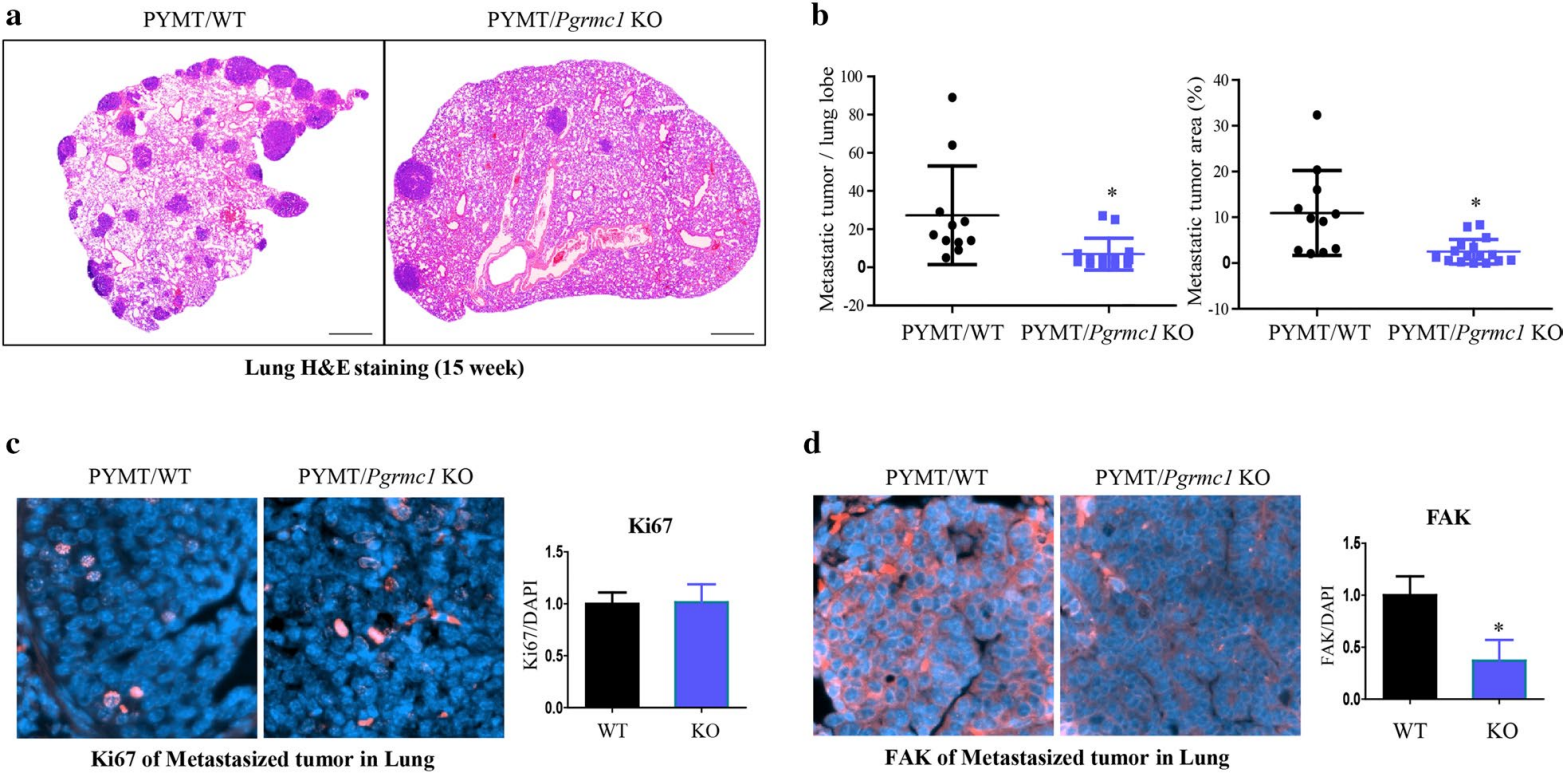
Genetic deletion of *Pgrmc1* suppresses the metastasis of breast cancer to the lungs. **a** H&E staining of the lungs of WT and *Pgrmc1* KO mice. Metastatic tumors are shown in deep purple color (scale bar: 800 µm). **b** Quantification of the number and the area of metastatic tumors per lung lobe. Image J was used for analysis. **c** Immunostaining of Ki67 in the metastatic tumors of WT and *Pgrmc1* KO mice. The number of Ki67-positive cells (pink) were counted. DAPI (blue) was used as an internal control. **d** Immunostaining of FAK in the metastatic tumors of WT and *Pgrmc1* KO mice. FAK-positive areas (pink) were quantified. DAPI (blue) was used as an internal control. Bars in the right panel show the mean ± standard deviation values. *, *p* < 0.05 versus WT mice

To investigate the status of metastasis according to the presence of *Pgrmc1*, the mice were sacrificed in the late phase of breast cancer at 15 weeks of age and their lungs were subjected to H&E staining (Fig. 4a). The number of metastatic tumors per lung lobe was significantly lower in *Pgrmc1* KO mice (*p* < 0.05, 23.7% compared with WT mice) (Fig. 4b, left panel). Likewise, *Pgrmc1* KO mice had a significantly smaller metastatic tumor area (*p* < 0.05, 19.6% compared with WT mice) (Fig. 4b, right panel). The proliferation rate of metastatic tumor, as measured by Ki67 immunostaining, was not significantly different between the two groups (Fig. 4c). Meanwhile, the expression of FAK was significantly decreased (*p* < 0.05, 36.9% compared with WT mice) in the metastatic tumors of *Pgrmc1* KO mice (Fig. 4d).

### Knock-down of PGRMC1 decreases the migratory ability of breast cancer cells

**Fig. 5:**
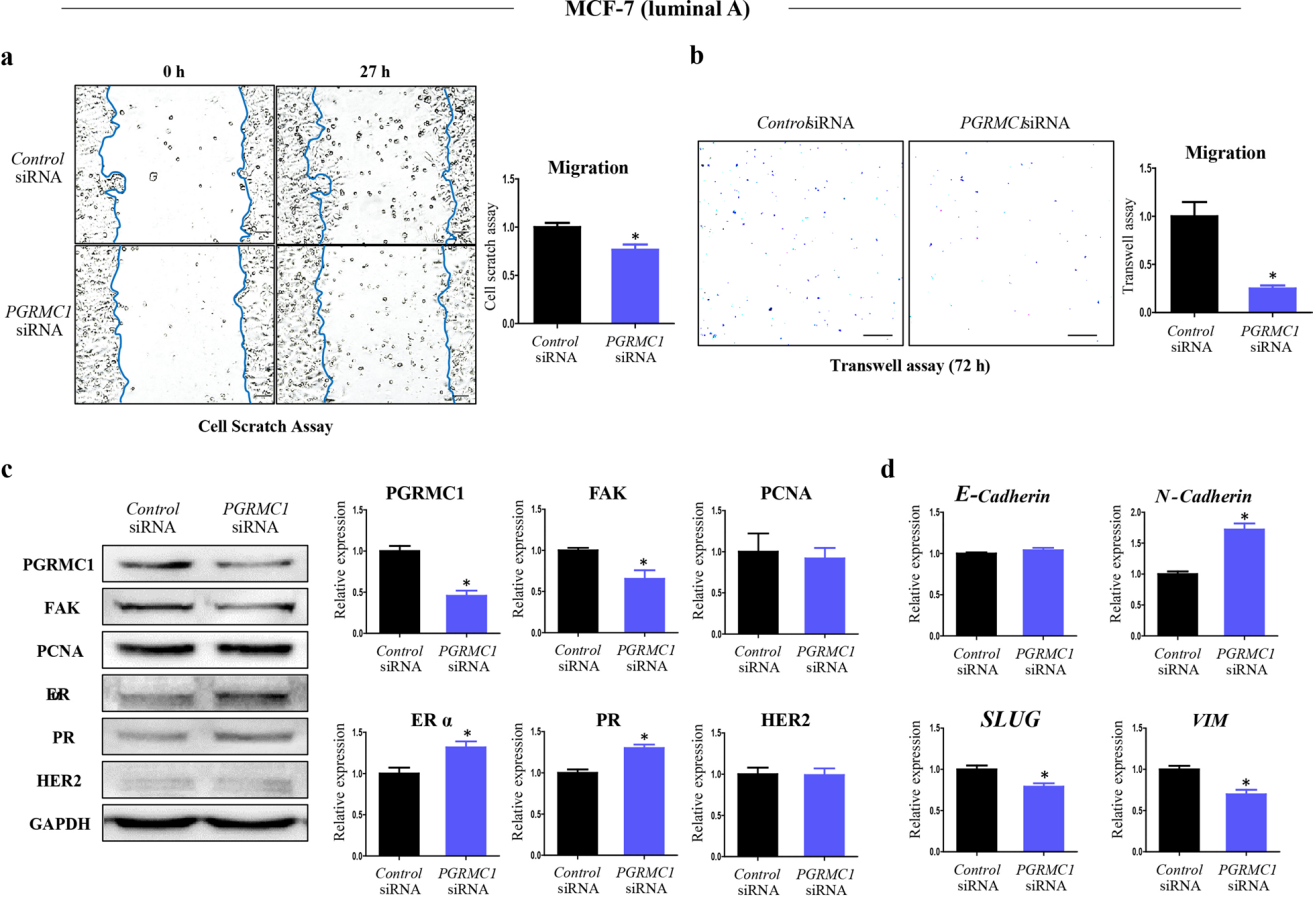
Knockdown of *PGRMC1* decreases the migratory ability of MCF-7 cells. **a** Cell scratch assay analysis and quantification of MCF-7 cells transfected with either *Control* siRNA or *PGRMC1* siRNA (scale bar: 25 µm). **b** Transwell analysis and quantification of MCF-7 cells transfected with either *Control* siRNA or *PGRMC1* siRNA (scale bar: 50 µm). **c** Western blot analysis and quantification of PGRMC1, FAK, PCNA, ERα, PR, and HER2 in MCF-7 cells transfected with either *Control* siRNA or *PGRMC1* siRNA. GAPDH was used as an internal control. **d** mRNA expression of an epithelial marker (*E-cadherin*) and mesenchymal markers (*N-cadherin, SLUG, VIM*) in MCF-7 cells transfected with either *Control* siRNA or *PGRMC1* siRNA. *Rplp0* was used as an internal control. Bars in the right panel show the mean ± standard deviation values. *p < 0.05 versus control siRNA

Several studies have investigated the relevance between PGRMC1 and migration in various types of cancers [18–21]; accordingly, we focused on how to detect the breast cancer cells undergoing EMT in culture systems. To evaluate the metastatic activity in vitro, both luminal A subtype breast cancer cells (MCF7 cells) and triple-negative breast cancer (MDA-MB-231 cells) were used in a series of experiments. We assessed how the suppression of PGRMC1 within breast cells affects their migration ability. We first cultured MCF7 cells in a medium depleted of sex steroids (i.e., containing 2% dextran charcoal-treated FBS), and performed cell scratch assay and transwell migration assay. Then, to examine whether the depletion of functional PGRMC1 in the MCF7 cells is directly responsible for the suppression of their migration ability, we knocked-down *PGRMC1* in MCF7 cells by treating them with a siRNA specific for the *PGRMC1* mRNA. Cell scratch assay showed decreased degree of cell migration in the *PGRMC1* siRNA group (*p* < 0.05, 76.7% compared with *Control* siRNA group) (Fig. 5a). The *PGRMC1* siRNA group also showed suppressed migratory ability in the transwell assay (*p* < 0.05, 24.9% compared with *Control* siRNA group) (Fig. 5b). PGRMC1 protein was decreased (*p* < 0.05, 45.7% compared with *Control* siRNA group) in the *PGRMC1* siRNA group (Fig. 5c).

Western blot analysis showed that FAK expression was decreased (*p* < 0.05, 65.5%) in the *PGRMC1* siRNA group (Fig. 5c). In contrast, the expression of proliferating cell nuclear antigen (PCNA), a component of cellular replication, did not show a significant difference between the two groups (Fig. 5c). Unexpectedly, the expression levels of ERα and PR were increased in the *PGRMC1* siRNA group (Fig. 5c). Among the mesenchymal markers, the level of *N-cadherin* was unexpectedly increased in the *PGRMC1* siRNA group, while those of *SLUG* and *VIM* were decreased in the *PGRMC1* siRNA group (*p* < 0.05, 79% and 69.7% compared with *Control* siRNA group, respectively) (Fig. 5d).

**Fig. 6:**
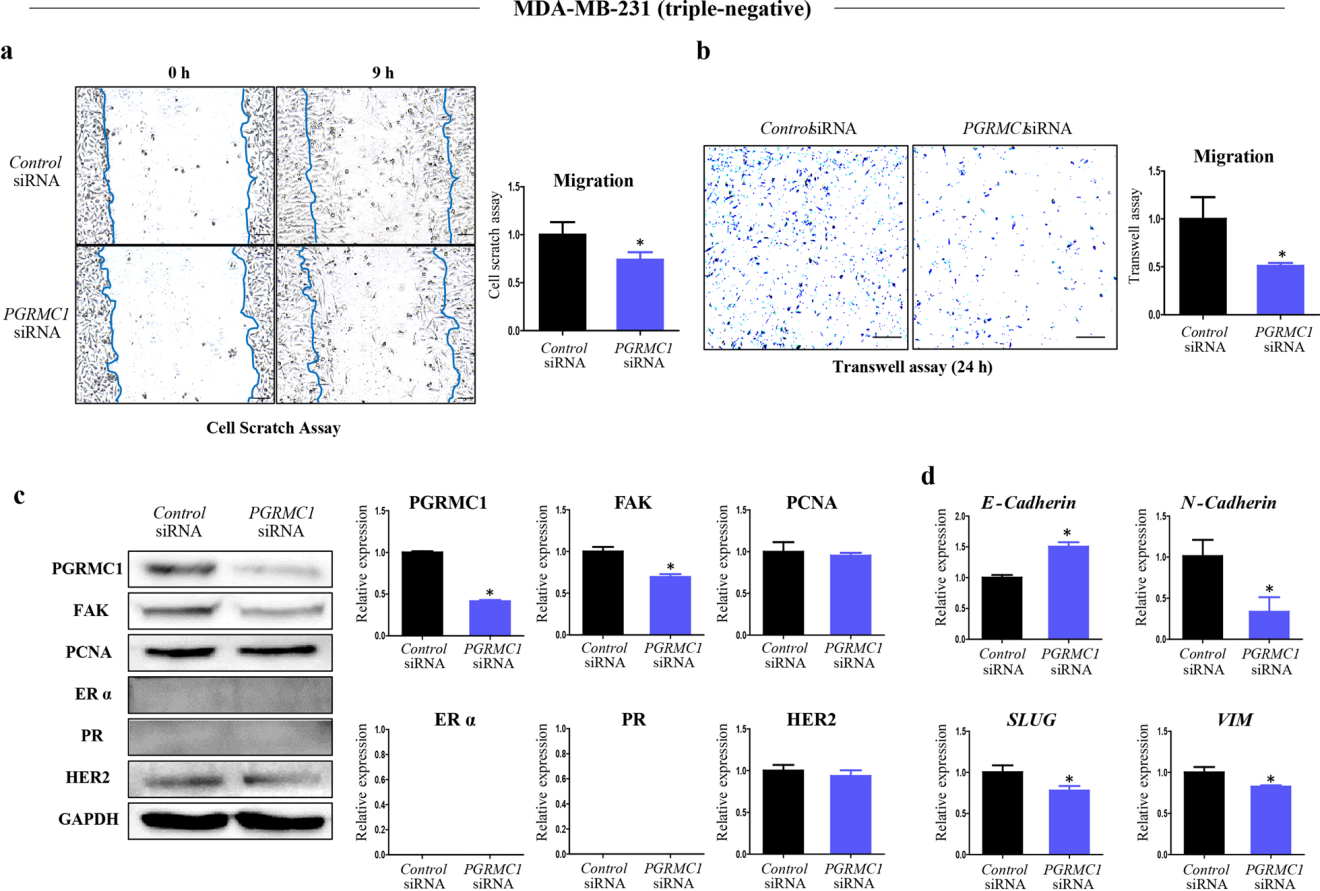
Knockdown of *PGRMC1* decreases the migratory ability of MDA-MB-231 cells. **b** Cell scratch assay analysis and quantification of MDA-MB-231 cells transfected with either *Control* siRNA or *PGRMC1* siRNA (scale bar: 25 µm). **b** Transwell analysis and quantification of MDA-MB-231 cells transfected with either *Control* siRNA or *PGRMC1* siRNA (scale bar: 50 µm). **c** Western blot analysis and quantification of PGRMC1, FAK, PCNA, ERα, PR, and HER2 in MDA-MB-231 cells transfected with either *Control* siRNA or *PGRMC1* siRNA. GAPDH was used as an internal control. **d** mRNA expression of an epithelial marker (*E-cadherin*) and mesenchymal markers (*N-cadherin, SLUG, VIM*) in MDA-MB-231 cells transfected with either *Control* siRNA or *PGRMC1* siRNA. *Rplp0* was used as an internal control. Bars in the right panel show the mean ± standard deviation values. *p < 0.05 versus control siRNA

Although PGRMC1 was shown to increase the proliferation of MDA-MB-231 cells in a xenograft model [22], the relationship between PGRMC1 and migration in MDA-MB-231 cells is unknown. To investigate whether the breast-specific regulation of *Pgrmc1* affects the migratory activity of triple-negative breast cancer cells, we used highly invasive MDA-MB-231 cells and found that transfection with *PGRMC1* siRNA also resulted in decreased cell migration in MDA-MB-231 cells according to cell scratch assay (*p* < 0.05, 74.1% compared with *Control* siRNA group) (Fig. 6a) and transwell assay (*p* < 0.05, 51% compared with *Control* siRNA) (Fig. 6b). When *PGRMC1* was knocked down, expression of PGRMC1 was suppressed (*p* < 0.05, 50.5%) in the *PGRMC1* siRNA group compared to the *Control* siRNA group (Fig. 6c). Similar to the results in MCF7 cells, the expression of FAK was suppressed in the *PGRMC1* siRNA group (*p* < 0.05, 69.4% compared with *Control* siRNA group) while that of PCNA was not significantly different between the two groups (Fig. 6c). Expression of ERα and PR was not observed in the MDA-MB-231 cells (Fig. 6c). The expression of *E-Cadherin* was increased by 1.51-fold in the *PGRMC1* siRNA group (*p* < 0.05) (Fig. 6d), while the expression levels of the mesenchymal markers *N-cadherin*, *SLUG*, and *VIM* were decreased in the *PGRMC1* siRNA group suppressed (*p* < 0.05, 33.1%, 77.8%, and 82.8% compared with *Control* siRNA group, respectively) (Fig. 6d). These results show that highly invasive triple-negative breast cancer cells with knockdown of *PGRMC1* showed similar changes in the EMT phenotypes to those of *Pgrmc1* KO mice.

## Discussion

Metastatic breast cancer is prone to recurrence and has a high mortality rate [23]. With advances in therapeutic approaches, the overall survival rate of patients with breast cancer has been improving in the past decades [24]. Nonetheless, drug resistance in metastatic cancer still exists [25], and a novel therapeutic target for metastatic cancer is needed. In our study using the PyMT transgenic murine breast cancer model, genetic deletion of *Pgrmc1* did not result in a significant difference in terms of cancer growth compared with WT mice. Nevertheless, the *Pgrmc1* KO mice showed a significantly lesser degree of lung metastasis, and we observed that the PGRMC1 protein is involved in FAK expression, EMT regulation, and cancer cell migration. Although the PyMT model cannot represent all types of breast cancer, our results are meaningful because lung metastasis in breast cancer is associated with high mortality; therefore, the suppression of PGRMC1 should be highlighted as a potential therapeutic strategy for increasing the life expectancy and survival rate of patients with breast cancer.

Hormone receptors including ER, PR, and HER2 are used for classifying the breast cancer subtypes into luminal A, luminal B, HER2-positive, and triple-negative. Considering that PGRMC1 was shown to be correlated with the ER status in breast cancer [11], PGRMC1 may have a role in the regulation of the progression of luminal A and luminal B subtypes of breast cancer. In a previous study using xenograft models, PGRMC1 depletion in MCF7 and T47D cells resulted in suppression of tumor growth [12]; as both MCF7 and T47D cells belong to the luminal A subtype, PGRMC1 was suggested to regulate the growth of breast cancer via ER signaling [12]. In the present study, *Pgrmc1* KO mice showed a slight decrease in tumor development along with a decrease in the expression of PR. However, as the difference in the size of WT and *Pgrmc1* KO tumors was not sufficient to show statistical significance, the development of breast cancer does not seem to be significantly affected by PGRMC1.

Although the cancer incidence and tumor growth were similar in both experimental groups, the survival duration was significantly longer in *Pgrmc1* KO mice than in WT mice, suggesting that the survival period was influenced by the degree of metastasis, which was significantly suppressed in *Pgrmc1* KO mice. To exclude the possible effect of other hormonal or paracrine interference, we starved the MCF7 cells and triple-negative MDA-MB-231 cells by incubation with charcoal dextran stripped-FBS; in line with the in vivo results, the knockdown of *Pgrmc1* in these cells resulted in the suppression of migration in the scratch assay and transwell assay. This is consistent with a previous study that observed the progression of mammary tumors by PGRMC1 in triple-negative breast cancer [18, 22]. Therefore, it can be speculated that PGRMC1 promotes the metastatic phenotype in breast cancer cells regardless of the interference hormone receptors. Furthermore, considering the recent evidence suggesting that the blood level of PGRMC1 is a potent marker for breast cancer prognosis [26], our study also shows the possibility of using *Pgrmc1* as a survival expectancy marker in patients with breast cancer.

Our study showed that *Pgrmc1* regulates the metastasis and migration of breast cancer cells, although the underlying mechanism of this phenomenon was not thoroughly delineated. Nonetheless, we observed several possible regulators (e.g., FAK, MMP9, EMT) that could be involved in the mechanism of metastatic induction by *Pgrmc1*. First, we observed that genetic deletion or silencing of *Pgrmc1* resulted in decreased expression of FAK, which was shown to negatively regulate invadopodia and positively regulate the invasion of breast cancer cells [27]. A recent study showed that the PGRMC1 protein is post-translationally modified or phosphorylated by progesterone [28], and that its phosphorylation influences the activation of FAK and alters the cellular migration depending on the phosphorylation site [29]. Also, similar to a previous study that demonstrated the lack of lung metastasis in FAK-deficient PyMT mice [30], the FAK expression was suppressed in *Pgrmc1* KO mice in this study.

Another possible mediator of the effect of PGRMC1 on breast cancer migration is MMP9, which promotes the colonization of the lungs by inducing metastatic aggressiveness as well as the migration and invasion of cancer cells while not significantly affecting the tumor burden [31]. Similar to MMP9, MMP2 also increases the invasion of breast cancer cells [32]. Notably, a previous study found that PGRMC1 regulates the activity and degradation of MMP9 [33], which is consistent with our data on the decreased expression of MMP9/MMP2 in the tumors of *Pgrmc1* KO mice.

Lastly, EMT may also be involved in the PGRMC1-induced migration of breast cancer cells. As an indicator of the motility of tumor cells, EMT is the process in which the morphology of tumor cells changes from cobblestone to spindle shape for the penetration of tumor capsule for migration and invasion [34]. In our study, the tumors of *Pgrmc1* KO mice showed a suppressed-EMT phenotype such as the induction of epithelial markers and suppression of mesenchymal markers. Moreover, MCF-7 cells decreased mesenchymal markers (*SLUG*, *VIM*) after the knockdown of PGRMC1. Furthermore, the EMT phenotype of MDA-MB-231 cells was suppressed in PGRMC1 knockdown.

In summary, the present study shows that while whole-body knockout of *Pgrmc1* was not sufficient for the suppression of growth of breast cancer, it significantly suppressed the degree of lung metastasis. Considering the previous findings on the association between ER and Pgrmc1, the slight decrease in the development of breast cancer in the *Pgrmc1* KO mice was unexpected. Importantly, *Pgrmc1* KO mice showed a significantly longer survival duration than did WT mice, which was likely due to the suppression of lung metastasis. In vitro analysis, *PGRMC1* also regulated the migration of both luminal A and triple-negative subtypes of breast cancer cells, especially in a steroid-starved condition. As metastatic breast cancer has a high mortality rate, *Pgrmc1* should be highlighted as a possible target for regulating tumor metastasis and increasing the survival expectancy of patients with breast cancer.

## Conclusions

Our experiments show that the decrease in the PGRMC1 protein leads to the suppression of metastasis in mice bearing breast cancer and migratory ability of MCF-7 and MDA-MB-231 breast cancer cells. These findings are particularly interesting considering that *Pgrmc1* KO mice showed extended survival compared with WT mice, without significant differences associated with the incidence and growth of tumors themselves. Therefore, these results provide evidence that the suppression of PGRMC1 in breast cancer cells could contribute to the reduction of lung metastasis via the regulation of FAK and EMT. The suppressive ability of PGRMC1 inhibition on tumor cell migration and metastasis is of potential clinical importance considering the critically high mortality rate of metastasis in breast cancer.

## Data Availability

The datasets used and/or analyzed during the current study are available from the corresponding author on reasonable request.

## Abbreviations

Pgrmc1: Progesterone receptor membrane component 1
ECM: Extracellular matrix
EMT: Epithelial–mesenchymal transition
FAK: Focal adhesion kinase
MMP: Matrix metallopeptidase
ERα: Estrogen receptor alpha
PR: Progesterone receptor
HER2: Human epidermal growth factor receptor-2
PCNA: Proliferating cell nuclear antigen
ERK: Extracellular regulated kinases
PARP: Poly(ADP-ribose) polymerase
